# Resurgence?

**DOI:** 10.1016/j.jacbts.2021.06.005

**Published:** 2021-07-26

**Authors:** Douglas L. Mann

Like many of you, I have been rejoicing as signs of normal life begin to emerge in my own community in Saint Louis, Missouri, as well as in other cities in the United States and all around the world. Every time I walk into a restaurant and see smiling, maskless faces enjoying the common bond of humanity, I am reminded that the resurgence of life that we are enjoying represents the incredible impact of translational science writ large. The collaborative efforts that enabled teams of scientists to develop multiple successful COVID-19 vaccines are highlighted in a special themed issue of *Nature* entitled “How to Collaborate: The Promise and Pitfalls of Partaking in Team Science” (1). This issue, which I highly recommend reading, features both the good, the bad, and some of the ugly parts of team science. As I read through the articles in the themed issue in *Nature*, I began to wonder what we as a community can learn from our current success, and how we can leverage this knowledge going forward.

## Alone Together

Translational scientists are joined together by a common bond of producing scientific results that directly benefit human health. Yet most scientists have grown up in an intensely competitive environment where they learned to compete for scant grant funding and work in isolation so that they would not be scooped by a competing lab or research group. How did scientists overcome these issues and learn to work together with unparalleled speed and effectiveness during the COVID-19 pandemic? The evolutionary history of *Homo sapiens* suggests that collaboration was essential to the success of our species in the past. More than 70,000 years ago, *Homo sapiens* learned to work together, to hunt collaboratively, and to forage for and process new foods. In his book *Sapiens: A Brief History of Humankind*, the historian and author Yuval Noah Harari (2) posits that *Homo sapiens* became the dominant human species because they developed the ability to collectively believe in the same fictive narratives, ideas, and goals, which enabled *sapiens* to cooperate on a much wider scale than any other species on Earth. Harari has termed this the Cognitive Revolution (2). One plausible explanation for why scientists collaborate is that it is part of our inherent nature to work together in order to survive. However, all one must do is watch the endless bickering between Republicans and Democrats during live coverage of the United States Congress on C-SPAN cable television, in order to realize that as a species, we are not inherently good at collaborating. In the themed issue in *Nature* that I mentioned (1), Lewis and Sadler (3) offer what I believe is a more satisfying explanation for how and why team science works. In their article, Lewis and Sadler describe how community–academic partnerships helped to deal with and overcome the water crisis in Flint, Michigan. They argue that: “The most important ingredient in making collaborations work is commitment: to producing research that is relevant, and to understanding many angles and perspectives. This means spending less time and attention on conventional metrics, such as published papers, journal impact factors and procured grants, and much more on nurturing relationships. In true community-based partnerships, a paper is incomplete without a link back to the local community” (3). The pandemic enabled industry–academic–government collaborations to occur in a way that could not ever have been imagined in a pre-pandemic world. The essential ingredient that led to the development of multiple safe and effective vaccines was the worldwide commitment to producing relevant research that linked back to communities.

## What’s in a Word?

I chose to entitle this Editor’s Page “Resurgence?” because the word has several different context-dependent meanings that are particularly germane to the post-pandemic world we are now entering. The English word *resurgent* (1808) is derived from the Latin prefix *re*- (anew, back, again) and the Latin word *resurgere* (to rise again, lift oneself, be restored). In this original context, resurgence means a rising again into life. In medical parlance, the word resurgence can also mean the return of a disease, as in the return of a virus in a community (eg, resurgence of polio or COVID-19 variants). Yuval Noah Harari wrote in *Homo Deus: A Brief History of Tomorrow*, the follow-on book to *Sapiens*, that “while we cannot be certain that some new Ebola outbreak or an unknown flu strain won’t sweep across the globe and kill millions, we will not regard it as an inevitable natural calamity… humankind has the knowledge and tools to prevent plagues, and if an epidemic nevertheless gets out of control, it is due to human incompetence rather than divine anger.” Harari believes that “we have the power to stop this, but so far we lack the necessary wisdom” (4,5). I also believe that we have the power to stop this; however, I also believe that the knowledge gained over the past 18 months may shine a light on a new path forward, one that is different than our current path, one that will allow us to acquire the requisite wisdom we will need to prevent the next global human tragedy. To accomplish this, we will need to study carefully what worked and what did not work. We will need to learn from our mistakes and commit to building upon our successes. And last, but certainly not least, rather than drift back into our own private scientific silos, we will need to nurture and expand upon collaborations between researchers, industry, and governments all across the globe. As always, I welcome your thoughts about what we can and should do things differently in the new post-pandemic world—either through social media (#JACC:BTS) or by email (jaccbts@acc.org).
